# Survival analysis of haematologic neoplasms in children and adolescents: a population-based study in a state of the Brazilian Legal Amazon

**DOI:** 10.3332/ecancer.2024.1764

**Published:** 2024-09-12

**Authors:** Paulo César Fernandes de Souza, Mariano Martinez Espinosa, Maria Teresa Bustamante Teixeira, Fernanda Cristina da Silva de Lima, Noemi Dreyer Galvão

**Affiliations:** 1 Instituto de Salud Colectiva de la Universidad Federal de Mato Grosso, Cuiabá, Mato Grosso 78060-900, Brasil; 2 Departamento de Salud del Estado de Mato Grosso, Cuiabá, Mato Grosso 78049-902, Brasil; 3 Departamento Salud Colectiva, Universidad Federal de Juiz de Fora, Juiz de Fora, Minas Gerais 36.036-900, Brasil; 4 División de Vigilancia y Análisis de Situación (DIVASI)/ Coordinación de Prevención y Vigilancia (Conprev) del Instituto Nacional del Cáncer (INCA), Río de Janeiro 20230-240, Brasil

**Keywords:** leukaemia, lymphoma, survival, information systems, child health

## Abstract

**Aims:**

To estimate the survival patterns of childhood leukaemias and lymphomas in Mato Grosso between 2001 and 2017.

**Methods:**

Retrospective population-based cohort study, with case information extracted from the population-based cancer registries (PBCRs) of Mato Grosso for the period 2001–2017. Cases aged 0–19 years diagnosed with microscopically confirmed leukaemias or lymphomas were eligible. Five-year relative survival was calculated using the Eldererer II method, considering the interval between diagnosis and death, loss to follow-up or censoring, after passive follow-up in the mortality information system. Cases registered only by death certificate were excluded.

**Results:**

510 cases of leukaemia were analysed, with a predominance of males (56.1%) and an age range of 0–4 years (34.9%). The 5-year relative survival rate was 77.3% (95% CI: 73.6;80.9). As for lymphomas, there were 261 cases, predominantly in males and in the age group 5–9 years. The 5-year relative survival rate was 84.7% (95% CI: 78.3;88.9), with a better prognosis for females and 87.7% (95% CI: 80.8;95.1) in the 5-9 years age group.

**Conclusion:**

The relative survival rates of childhood leukaemia and lymphoma in the state of Mato Grosso were lower than those of developed countries. The importance of early diagnosis and timely treatment for better outcomes is highlighted. The importance of using and continuously improving the quality of information from PBCRs in the state of Mato Grosso is highlighted.

## Introduction

Trends in cancer survival at the population level, as assessed through population-based cancer registries (PBCRs), play an essential role in assessing progress in disease control. On the other hand, information derived from hospital registries or clinical trials is generally limited to a specific cohort of patients, is susceptible to bias, and cannot provide a comprehensive assessment of population-wide survival rates [1, 2].

Cancer survival is an important indicator for evaluating cancer control efforts. Generally, interpretations of survival study analyses are complex and different methods can help to understand the magnitude of the disease. Relative survival, a method widely used in analyses based on PBCR data, consists of the probability that an individual remains alive for a given period after cancer diagnosis compared with the experience of the general population, generally using the population life table to calculate the rate [3, 4].

Information from the Brazilian PBCRs has previously shown, using an indirect measure of the mortality/incidence ratio, that the average survival rates for children and adolescents diagnosed with leukaemias and lymphomas were 40% and 81%, respectively, between 2003 and 2007 [5].

Several factors contribute to global disparities in childhood cancer outcomes, including inequalities in access to diagnosis, treatment and palliative care, human resource shortages, financial barriers and diagnosis at a later stage in low- and middle-income countries [6].

This study presents, for the first time, a long-term analysis of estimates of relative survival rates for childhood leukaemia and lymphoma in Mato Grosso, using information from the Mato Grosso PBCRs from 2001 to 2017.

## Methodology

This was a population-based retrospective cohort study. Information on children and adolescents (aged 0–19 years) diagnosed with leukaemias or lymphomas was provided by the PBCRs of Mato Grosso. The state of Mato Grosso is located in the Midwest region of Brazil and is part of the Legal Amazon, which covers an area of 5,015,146.008 km^2^, corresponding to 58.93% of the national territory [7]. Cases of leukaemia and lymphoma were grouped according to the International Classification of Pediatric Cancer (ICCC). The leukaemias were: I.a. acute lymphoblastic leukaemia (ALL); I.b. Acute myeloid leukaemia (AML); I.c. Chronic myeloproliferative diseases; I.d. Myelodysplastic syndromes and other myeloproliferative diseases; I.e. Specified and unspecified leukaemias. The lymphomas were: II.a. Hodgkin's lymphoma (HL); II.b. Non-Hodgkin's lymphoma (except Burkitt's lymphoma (BL)) (NHL); II.c. BL; II.d. Miscellaneous lymphoreticular neoplasms and II.e. Unspecified lymphomas [8].

Eligible cases from 2001 to 2017 were followed through 31 December 2022. Vital status was obtained passively by linking the PBCR incidence database with death records from the state mortality information system. Patients not found in the database at the final follow-up date (12/31/2022) were considered alive.

Cases diagnosed by death certificate only (DCO) of leukaemias (*n* = 47, 8.4%) and lymphomas (*n* = 5, 1.9%) were excluded from the survival analysis. Survival time was determined by calculating the interval between the dates of diagnosis and death (failure) or the date of the last follow-up contact (censoring) over 5 years. Age at diagnosis was classified into 4 groups: 0–4, 5–9, 10–14 and 15–19 years. Absolute and relative frequencies of sociodemographic and clinical variables were described, together with their respective confidence intervals.

Five-year relative survival was calculated by the Edererer II method, using the ratio between observed survival and expected survival for individuals in the general population, according to age, sex, leukaemia or lymphoma subtype and place of residence (Capital = Cuiabá, Várzea Grande and other municipalities = 139 municipalities in the state) [9].

Expected survival was derived from the general population mortality table using the annual survival probabilities published by the Brazilian Institute of Geography and Statistics [10] in 2010 for the state of Mato Grosso.

Estimators of the observed survival probability were calculated using the Kaplan–Meier method and the log-rank test was used to test for differences between survival curves in successive periods, considering a significance level of 5% [11].

All statistical analyses were performed with R Studio version 4.3.0. The study was duly approved by the Ethics Committee on Human Research in the Health Area of the UFMT CAAE: 63509222.6.0000.8124, opinion number: 5.709.469 and by the Research Ethics Committee of the Health Secretariat of the State of Mato Grosso (SES-MT) CAAE:63509222.6.3001.5164 opinion number 5.779.146.

## Results

A total of 771 new cases of leukaemia and lymphoma were registered between 2001 and 2017 in Mato Grosso, excluding OCD cases (52 cases). Leukaemias were the most common, with 510 new cases, and were more frequent in males (56.1%), in the age group 0–4 years (34.9%) and in black-skinned patients (59.6%). The most frequent subtype was ALL, with 75.3% of cases. About 35.1% of patients lived in the state capital and 64.9% in other municipalities. Regarding the quality of information from the PBCRs of Mato Grosso, 91.6% of the cases were diagnosed by microscopic verification (MV), while 8.4% were classified as DCO. During the study period, 47.8% of the patients died (Table 1). Regarding lymphomas, 261 cases were diagnosed, with higher frequency in male sex (63.6%), age between 5 and 9 years (31.8%), black skin color (50.6%), with HL (42.1%), residents in other municipalities of the state of Mato Grosso (68.2%) and 33.3% died (Table 1).

The 5-year relative survival rate for all leukaemia subtypes was 77.3% and increased over the period, with 49.7% between 2001 and 2008 and 75.0% between 2009 and 2017, data not shown in the table. Survival rates among age groups showed a worse prognosis with increasing age, with the highest survival rates observed in patients aged 0–4 years (80.1%) and in lymphoid leukaemias (80.0%). No significant differences were found between the variables analysed, except for leukaemia subtypes (Table 2).

The 5-year relative survival probability for all lymphoma subgroups was 84.7% and increased between 2001 and 2008 and between 2009 and 2017, from 50.0% to 85.5%, respectively, data not shown in the Table. The highest relative survival rates were observed in female patients (87.4%), aged 5–9 years (87.7%) and with HL (93.5%). There were no significant differences between variables, except for lymphoma subtypes (*p* = 0.0001), as shown in Table 3.

**Figure 1.**
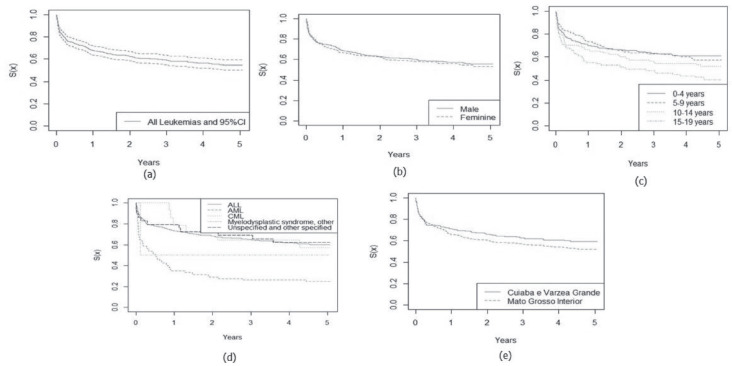
Five-year relative survival curves according to all leukaemias (a) and sex (b), age (c), leukaemia subtype (d) and city (e) in Mato Grosso, Brazil, from 2001 to 2017.

**Figure 2.**
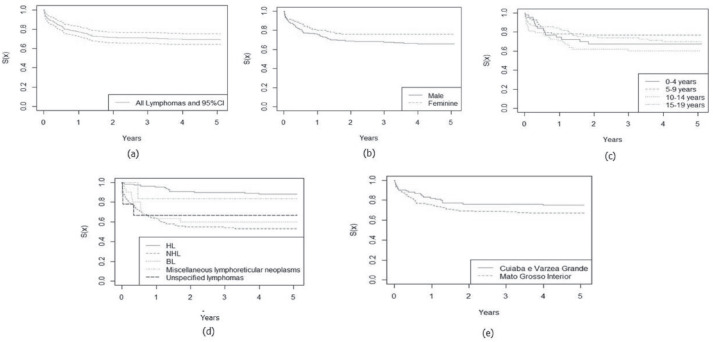
Five-year relative survival curves according to all lymphomas (a) and sex (b), age (c), leukaemia subtype (d) and city (e) in Mato Grosso, Brazil, from 2001 to 2017.

In Figures 1 and 2, the estimated relative survival curves for all leukaemias and lymphomas are presented according to sex, age, subtypes and place of residence.

## Discussion

Using data from the Mato Grosso PBCRs, we reported the 5-year relative survival for leukaemias and lymphomas in children and adolescents aged 0–19 years in Mato Grosso between 2001 and 2017. We observed that more than 91.6% of leukaemia cases and 98.1% of lymphomas were histologically verified, indicating the reliability of the Mato Grosso PBCR data. Regarding 5-year relative survival, the estimate was 76.3% for leukaemias and 83.7% for lymphomas.

In addition, there was an increase in relative survival estimates during the period analysed, with significant gains among age groups and leukaemia and lymphoma subgroups. To the best of our knowledge, this study is the first to present relative survival estimates for leukaemia and lymphoma in children and adolescents based on data from the PBCRs of Mato Grosso.

Regarding the quality of information, the percentage of cases registered only by death certificate was 8.4% for leukaemias and 1.9% for lymphomas, percentages much higher than those found in the USA and the European median [12]. These high percentages point to the need to improve the collection of information by the PBCRs and to institute an active search for cases within its area of action, to allow investigation and rescue of these cases.

The 5-year relative survival rate for leukaemias in the population aged 0–19 years in Mato Grosso (77.3%) was lower than that of developed countries. According to the results of the CONCORD 3 study, disparities in 5-year net survival estimates for ALL leukaemia between 2010 and 2014 were identified, ranging from 49.8% in Ecuador to 95.2% in Finland. Even with advances in childhood cancer treatment, middle-income countries have the lowest survival probabilities, often below 30%. Unfortunately, in these countries, cancer is detected at advanced stages and with little chance of cure or effective treatment. The study also revealed that, for lymphomas, variations in 5-year survival rates were lower compared to ALL in the 0–14 age group. Specifically, in Brazil, there was an improvement in survival of 69.2% between 2000 and 2004, increasing to 88.2% between 2010 and 2014. The authors suggest that these results may reflect timely access to diagnosis and treatment [13].

Disparities in net survival estimates by subtype were identified [14] for the periods 1995 to 1999 and 2005 to 2009 in children aged 0–14 years. The study showed a more favourable prognosis for children aged 1–4 years. Recently [15], other studies corroborated our results by showing that, between 2010 and 2014, 5-year net survival was higher in patients aged 0–19 years and 20–24 years diagnosed with ALL compared with AML. Survival was longer in children compared to adolescents and young adults.

For lymphomas, survival rates in children aged 0–14 years can vary considerably in different regions of the world, as indicated by the results of the CONCORD 3 study, which estimated net survival in 29 countries and found values above 90%, with a range of 80%–95% between 2010 and 2014 [13].

A recent study [16] showed a remarkable increase in 5-year relative survival estimates in Australia, which were 82.2% between 1995 and 2006, and from 2007–2016, reaching 90.4%, covering the NHL subgroup. However, when comparing the survival estimates for NHL found in our results with those presented by the Australian study, we note that the 5-year relative survival found in our study (84.7%) was higher than those reported by the authors in the 1983–1995 and 1995–2006 periods, which were (76.8% and 82.2%).

Other studies have shown that survival estimates above 85% for leukaemias and lymphomas have led to a considerable reduction in mortality rates, as reported in the USA, Europe, Latin America, Asia and Oceania [17–19]. Despite considerable therapeutic advances that have had a global impact on increasing survival, high mortality remains a challenge in lower-middle income countries, where the probability of survival often remains below 30% [20].

In Brazil, survival studies based on information from the PBCRs in children under 15 years of age conducted in Goiânia (1989-94) and São Paulo (1993, 1997-98) revealed survival estimates for leukaemias and lymphomas of 27% and 67%, respectively, in Goiânia. In the city of São Paulo, the probability of survival was 41% and 66%, respectively [21, 22]. In Recife [23], the relative survival rate for leukaemias was 69.8%. Another study in Goiânia found a relative survival of 72.7% for lymphomas (Group II) and 56.6% for leukaemias (Group I) [24].

Silva and Latorre [25] estimated the survival of children aged 0–14 years with ALL in the municipality of São Paulo between 1997 and 2008 and found an overall survival probability of 68%. In a referral centre for pediatric tumours in Recife, the 5-year overall survival rate was 70% for NHL, with the Burkitt type being the most prevalent and no clinical or sociodemographic variable was associated with the probability of patient survival [26].

In 2016, the National Cancer Institute [5] published information on cancer incidence, mortality and hospital morbidity in children, adolescents and young adults in Brazil, estimating a median survival of 60% for leukaemias and 81% for lymphomas. It should be noted that the survival estimates observed in our study for leukaemias (77.3%) and lymphomas (84.7%) were higher than the rates in Brazilian studies and should be interpreted with caution due to regional differences in access and organisation of health services in the regions of the country.

It should be noted that, unlike adult cancers, childhood cancer is rare and its etiology is heterogeneous, making it difficult to identify risk factors. Genomic and case-control studies have provided answers, reducing the gaps in knowledge about the disease. Environmental risk factors remain a challenge for childhood cancer epidemiology, whereas associated factors such as birth weight, parental age, congenital anomalies and genetic disorders are well established [27].

There were an estimated 397,000 new cases of cancer in children and adolescents aged 0–19 years worldwide in 2015. However, this is likely to be a significant underestimate, as about 224,000 cases were diagnosed and 172,000 went undiagnosed, representing a difference of 43% worldwide. The burden is considerable, as childhood cancer is the sixth leading cause of the total cancer burden and the ninth leading cause of childhood disease burden worldwide. Fewer than four in ten children diagnosed with cancer are cured, despite cure rates in high-income countries more than doubling [28, 29].

Late or secondary effects in the treatment of childhood cancer are important factors in understanding and intervening in the magnitude of the burden of disease borne by survivors following a diagnosis of childhood cancer [30]. This seems to suggest that patterns of inequality and their implicit mechanisms differ between adult cancer and between types of childhood cancer.

Survival following a diagnosis of childhood cancer is influenced by a number of elements, including the patient's age group, the nature of the neoplasm, the stage of the disease at diagnosis and other clinical parameters. In addition, variables related to the healthcare system, such as accessibility to contemporary, high-quality medical care, play a crucial role in determining outcomes [31, 32].

A systematic review that evaluated the determinants of delayed diagnosis and care of childhood cancer in low- and middle-income countries identified a combination of cultural, socioeconomic and demographic factors, such as the use of traditional medicine, socioeconomic and demographic inequalities (such as low parental education and income and rural populations). However, the results of the review failed to establish an association between these factors and childhood cancer mortality, suggesting further studies to assess how these factors affect survival [33].

In this context, in 2018 WHO launched a new initiative with the goal of increasing childhood cancer survival rates to at least 60% worldwide by 2030, saving more than 1 million children and simultaneously improving the quality of life of all patients. Addressing childhood cancer is part of the implementation of the resolution on cancer, adopted in May 2017 by the Health Assembly, which mandates governments and WHO to accelerate action to achieve the specific goals of the United Nations Global Plan of Action and the 2030 Agenda for Sustainable Development to reduce premature mortality from câncer [34].

The strengths of the study relate to the population coverage of the Mato Grosso PBCRs,, which virtually covers the entire state, and the high quality of the data, allowing stratified analysis by subtype. This study provides a solid basis for future research on clinical details, socioeconomic determinants and their potential impact on survival, given that inequalities persist between and within countries. In addition, the analysis of relative survival, as well as the PBCR time series, allowed for international comparisons and robust information, given that most survival studies are based on clinical trials and are hospital-based. Despite the different types of methodologies for estimating survival, the discrepancy between the rates of the different methods is smaller in pediatric cancers compared to adult cancers, given that the concurrent causes of death are higher in the adult population [35].

Limitations of the study include: information on the staging variable, which is an important predictor variable for understanding cancer survival. This variable is not systematically collected by PBCRs, especially in low- and middle-income countries. There is a need to improve the follow-up of patients diagnosed with cancer, assessing survival and associated factors, such as the rate of treatment abandonment [31].

## Conclusion

Our analyses showed that 5-year relative survival rates for childhood leukaemias and lymphomas in Mato Grosso were below those of developed countries. Although challenges remain, these findings emphasize the importance of early diagnosis and timely treatment strategies, as well as improving the quality of cancer registry information. Continued efforts to reduce disparities in medical care and the implementation of public health policies are crucial to further improve outcomes for these diseases in pediatric patients.

## List of abbreviations

ALL, Acute lymphoblastic leukaemia; DCO, Death certificate only; ICCC-3, International Classification of Pediatric Cancer; LH, Hodgkin's lymphoma; NHL, Non-Hodgkin's Lymphoma; PBCRs, Population-based cancer registries; VM, Microscopic verification.

## Conflicts of interest

Nothing to declare.

## Funding

There was no source of funding for the study.

## Author contributions

PCFS conceived the study, developed the data management, exploitation and analysis. MME, FCSL, NDG and MTBT contributed to the draft versions of the article. All authors contributed to the critical review and approval of the final version and agreed to be responsible for all aspects of the work.

## Enrollment to the study

Identification/approval number by the Committee of Ethics in Research with Human Beings in the Health Area – CEP of the Federal University of Mato Grosso – UFMT, opinion number: 5.709.469, and by the Committee of Ethics in Research of the Health Secretariat of the State of Mato Grosso, opinion number: 5.779.146.

## Figures and Tables

**Table 1.** Description of sociodemographic and clinical characteristics according to ICCC-3 diagnostic group in children and adolescents (0–19 years) with leukaemias and lymphomas in Mato Grosso, Brazil, from 2001 to 2017.

Variables	leukaemias		Lymphomas	
	*n*	%	*n*	%
Sex				
Male	286	56.1	166	63.6
Feminine	224	43.9	95	36.4
Idad group				
00–04 years	178	34.9	43	16.5
05–09 years	145	28.4	83	31.8
10–14 years	99	19.4	59	22.6
15–19 years	88	17.3	76	29.1
Breed color				
Blanca	157	30.8	82	31.4
Black	304	59.6	132	50.6
Indigenous	3	0.6	2	0.8
No information	46	9	45	17.2
Cancer groups according to *ICCC-3				
Ia. Acute lymphoblastic leukaemia (ALL)	384	75.3	-	-
Ib. AML	80	15.7	-	-
Ic. Chronic myeloproliferative diseases	14	2.7	-	-
Id. Myelodysplastic syndrome and other myeloproliferative disorders	2	0.4	-	-
Ie. Unspecified leukaemias and other specified leukaemias.	30	5.9	-	-
Place of residence				
Capital (Cuiabá and Várzea Grande)	179	35.1	83	31.8
Other municipalities in the state of Mato Grosso	331	64.9	178	68.2
Information quality PBCRs				
Microscopic verification (MV)	510	91.6	261	98.1
By death certificate only (DCO)	47	8.4	5	1.9
Vital status				
Alive	266	52.2	174	66.7
Dead	244	47.8	87	33.3
Cancer groups according to *ICCC-3				
IIa. HL	-	-	110	42.1
IIb. NHL (except BL)		-	106	40.6
IIc. BL	-	-	30	11.5
IId. Various lymphoreticular neoplasms	-	-	6	2.3
IIe. Unspecified lymphomas	-	-	9	3.4

* ICCC-3: International Classification of Pediatric Cancer

**Table 2.** Five-year relative survival according to sex, age, ICCC-3 leukaemia diagnosis group and place of residence in Mato Grosso, Brazil, from 2001 to 2017.

Variables	Relative survival (%) 95%CI	Log-rank test (*p*)
Geral	77.3 (73.6;80.9)	
Sex		
Male	77.8 (64.6;81.7)	0.60
Feminine	76.5 (63.7;80.8)	
Idad group		
0–4 years	80.1 (68.3;84.2)	0.01
5–9 years	78.3 (69.65;84.7)	
10–14 years	74.5 (59.8;81.6)	
15–19 years	69.6 (55.7;77.8)	
Cancer groups according to ICCC-3 leukaemias		
Ia. Acute lymphoblastic leukaemia (ALL)	80.0 (67.8;82.6)	<0.0001*
I.b AML	61.9 (52.2;73.4)	
Ic. Chronic myeloproliferative diseases	75.1 (56.2;100)	
Id. Myelodysplastic syndrome and other myeloproliferative diseases	50.0 (18.8;100)	
Ie. Unspecified and other specified leukaemias.	79.3 (66.1;95.2)	
Place of residence		
Capital (Cuiabá and Várzea Grande)	78.5 (80.5;81.5)	0.20
Other municipalities in the state of Mato Grosso	75.7 (61.6;79.5)	

* Statistically significant (*p* < 0.05); 95%CI: confidence interval; ICCC-3: International Classification of Pediatric Cancer

**Table 3.** Five-year relative survival according to sex, age, CICI-3 lymphoma diagnostic group and place of residence in Mato Grosso, Brazil, from 2001 to 2017.

Variables	Relative survival (%)	Log-rank test (p)
Geral	84.7 (78.3;88.9)	
Sex		
Male	82.5 (77.0;88.6)	0.06
Feminine	87.4 (81.0;94.3)	
Idad group		
0–4 years	83.8 (73.5;95.4)	0.30
5–9 years	87.7 (80.8;95.1)	
10–14 years	79.3 (69.6;90.3)	
15–19 years	84.2 (76.5;92.8)	
Cancer groups according to ICCC-3 Lymphomas		
II.a HL	93.5 (89.0;98.3)	<0.0001*
II.b NHL (except BL)	76.0 (68.3;84.5)	
IIc. BL	78.4 (65.2;94.3)	
II.d Miscellaneous lymphoreticular neoplasms	83.4 (60.1;100)	
II.e Unspecified lymphomas	77.8 (56.0;100)	
City of residence		
Capital (Cuiabá and Várzea Grande)	86.8 (79.8;94.3)	0.10
Other municipalities in the state of Mato Grosso	83.2 (77.8;88.9)	

* Statistically significant (*p* < 0.05); 95%CI: confidence interval; ICCC-3: International Classification of Pediatric Cancer

## References

[1] Parkin DM (2008). The role of cancer registries in cancer control. Int J Clin Oncol.

[2] Piñeros M, Méry L, Soerjomataram I (2021). Scaling up the surveillance of childhood cancer: a global roadmap. J Natl Cancer Inst.

[3] Cho H, Howlader N, Mariotto AB (2011). Estimating relative survival for cancer patients from SEER using expected rates based on Edererer I versus Ederer II method. Surveillance Research Program, National Cancer Institute, Technical Report, 1.

[4] Ellis L, Woods LM, Estève J (2014). Cancer incidence, survival and mortality: explaining the concepts. Int J Cancer.

[5] de Oliveira Santos M (2016). Incidência, mortalidade e morbidade hospitalar por câncer em crianças, adolescentes e adultos jovens no Brasil: informações dos registros de câncer e do sistema de mortalidade.

[6] Atun R, Bhakta N, Denburg A (2020). Sustainable care for children with cancer: a Lancet Oncology Commission. Lancet Oncol.

[7] Instituto Brasileiro de Geografia e Estatística (2023). Amazônia Legal.

[8] Steliarova-Foucher E, Stiller C, Lacour B (2005). International classification of childhood cancer, third edition. Cancer.

[9] Ederer F, Axtell LM, Cutler SJ (1961). The relative survival rate: a statistical methodology. Natl Cancer Inst Monogr.

[10] Instituto Brasileiro de Geografia e Estatística (2013). Tábuas abreviadas de mortalidade por sexo e idade, 2010.

[11] Kaplan EL, Meier P (1958). Nonparametric estimation from incomplete observations. J Am Stat Assoc.

[12] Steliarova-Foucher E, Colombet M, Ries LAG (2017). International incidence of childhood cancer, 2001–10: a population-based registry study. Lancet Oncol.

[13] Allemani C, Matsuda T, Di Carlo V (2018). Global surveillance of trends in cancer survival 2000–14 (CONCORD-3): analysis of individual records for 37,513,025 patients diagnosed with one of 18 cancers from 322 population-based registries in 71 countries. Lancet.

[14] Bonaventure A, Harewood R, Stiller CA (2017). Worldwide comparison of survival from childhood leukaemia for 1995–2009, by subtype, age, and sex (CONCORD-2): a population-based study of individual data for 89,828 children from 198 registries in 53 countries. Lancet Haematol.

[15] Ssenyonga N, Stiller C, Nakata K (2022). Worldwide trends in population-based survival for children, adolescents, and young adults diagnosed with leukaemia, by subtype, during 2000–14 (CONCORD-3): analysis of individual data from 258 cancer registries in 61 countries. Lancet Child Adolesc Health.

[16] Youlden DR, Baade PD, Moore AS (2023). Childhood cancer survival and avoided deaths in Australia, 1983–2016. Paediatr Perinat Epidemiol.

[17] Bosetti C, Bertuccio P, Chatenoud L (2010). Childhood cancer mortality in Europe, 1970–2007. Eur J Cancer.

[18] Chatenoud L, Bertuccio P, Bosetti C (2010). Childhood cancer mortality in America, Asia, and Oceania, 1970 through. Cancer.

[19] Siegel L, Miller KD, Jemal A (2020). Cancer statistics. CA Cancer J Clin.

[20] Lam C, Howard SC, Bouffet E (2019). Science and health for all children with cancer. Science.

[21] Braga PE, Latorre MRD, Curado MP (2002). Câncer na infância: análise comparativa da incidência, mortalidade e sobrevida em Goiânia (Brasil) e outros países. Cad Saúde Pública.

[22] Mirra AP, Latorre MRDO, Veneziano DB (2004). Incidência, mortalidade e sobrevida do câncer da infância no município de São Paulo.

[23] Lins MM, Santo MO, Albuquerque MFPM (2017). Incidence and survival of childhood leukemia in Recife, Brazil: a population-based analysis. Pediatr Blood Cancer.

[24] Oliveira MM, Silva DRM, Ramos FR (2020). Children and adolescents cancer incidence, mortality and survival a population-based study in Midwest of Brazil. Cancer Epidemiol.

[25] Silva FF, Latorre MRDO (2020). Sobrevida das leucemias linfoides agudas em crianças no Município de São Paulo, Brasil. Cad Saúde Pública.

[26] Pedrosa MF, Pedrosa F, Lins MM (2007). Non-Hodgkin's lymphoma in childhood: clinical and epidemiological characteristics and survival analysis at a single center in Northeast Brazil. J Pediatr (Rio J).

[27] Spector LG, Pankratz N, Marcotte EL (2015). Genetic and nongenetic risk factors for childhood cancer. Pediatr Clin North Am.

[28] GBD 2017 (2019). The global burden of childhood and adolescent cancer in 2017: an analysis of the Global Burden of Disease Study 2017. Lancet Oncol.

[29] Ward ZJ, Yeh JM, Bhakta N (2019). Estimating the total incidence of global childhood cancer: a simulation-based analysis. Lancet Oncol.

[30] Landier W, Armenian S, Bhatia S (2015). Late effects of childhood cancer and its treatment. Pediatr Clin North Am.

[31] Bhakta N, Force LM, Allemani C (2019). Childhood cancer burden: a review of global estimates. Lancet Oncol.

[32] Erdmann F, Frederiksen LE, Bonaventure A (2021). Childhood cancer: survival, treatment modalities, late effects and improvements over time. Cancer Epidemiol.

[33] Cotache-Condor C, Kantety V, Grimm A (2023). Determinants of delayed childhood cancer care in low- and middle-income countries: a systematic. PediatrBlood Cancer.

[34] World Health Organization (2019). Global Initiative for Childhood Cancer.

[35] Soliman RM, Elhaddad A, Oke J (2020). Temporal trends in childhood cancer survival in Egypt, 2007 to 2017: a large retrospective study of 14,808 children with cancer from the Children's Cancer Hospital Egypt. Int J Cancer.

